# Diabetes and Cognitive Deficits in Chronic Schizophrenia: A Case-Control Study

**DOI:** 10.1371/journal.pone.0066299

**Published:** 2013-06-20

**Authors:** Mei Han, Xu-Feng Huang, Da Chun Chen, Meihong Xiu, Thomas R. Kosten, Xiang Yang Zhang

**Affiliations:** 1 Centre for Translational Neuroscience, School of Health Sciences, IHMRI, University of Wollongong, Wollongong, New South Wales, Australia; 2 Schizophrenia Research Institute, Sydney, New South Wales, Australia; 3 Beijing HuiLongGuan Hospital, Peking University, Beijing, PR China; 4 Menninger Department of Psychiatry and Behavioral Sciences, Baylor College of Medicine, and Michael E. DeBakey VA Medical Center, Houston, Texas, United States of America; Chiba University Center for Forensic Mental Health, Japan

## Abstract

Cognitive impairment occurs in both schizophrenia and diabetes. There is currently limited understanding whether schizophrenia with diabetes has more serious cognitive deficits than schizophrenia without diabetes or diabetes only. This study assessed cognitive performance in 190 healthy controls, 106 diabetes only, 127 schizophrenia without diabetes and 55 schizophrenia with diabetes. This study was conducted from January 2008 to December 2010. Compared to healthy controls, all patient groups had significantly decreased total and five index RBANS scores (all p<0.01–p<0.001), except for the visuospatial/constructional index. Schizophrenia with diabetes performed worse than schizophrenia without diabetes in immediate memory (p<0.01) and total RBANS scores (<0.05), and showed a trend for decreased attention (p = 0.052) and visuospatial/constructional capacity (p = 0.063). Schizophrenia with diabetes performed worse than diabetes only in immediate memory (p<0.001) and attention (p<0.05), and showed a trend for decreased total RBANS scores (p = 0.069). Regression analysis showed that the RBANS had modest correlations with schizophrenia’ PANSS scores, their duration of current antipsychotic treatment, and diagnosis of diabetes. Schizophrenia with co-morbid diabetes showed more cognitive impairment than schizophrenia without diabetes and diabetes only, especially in immediate memory and attention.

## Introduction

Cognitive deficits in schizophrenia have been well identified and are important indices of functional and treatment outcomes in patients [1], [2], [3]. Cognitive impairment in schizophrenia involves multiple domains, such as memory, attention, perception and processing speed [4], [5], [6]. Longitudinal studies have suggested that many of these cognitive impairments are stable over time and may persist after the remission of psychotic symptoms [7], [8]. The deficits in cognitive function can decrease the quality of life and increase unemployment rates for patients with schizophrenia [9].

Diabetes is a complex endocrine disease with a series of complications. Human and animal studies show that diabetes is also associated with cognitive impairment [10], [11], [12]. Diabetes related factors such as insulin resistance, hyperglycemia, hypertension and lipid metabolic disorders may affect cognitive function [13], [14], [15]. Moreover, studies have suggested that verbal memory and processing speed are the cognitive domains which are usually impaired in diabetes [16], [17], [18].

Diabetes has been reported to be about two to four times higher in patients with schizophrenia than in the general population, and it occurs in about 10–15% of patients with schizophrenia [19], [20], [21]. As schizophrenia or diabetes alone has cognitive vulnerability, the co-morbidity of both diseases may lead to an increased rate and magnitude of cognitive deficits. However, the contribution of diabetes to cognitive impairment in schizophrenia has seldom been investigated. To our knowledge, only two studies addressed this issue, with a preliminary result that schizophrenia with diabetes was more cognitively impaired than schizophrenia without diabetes [17], [22]. Moreover, the severity of these cognitive impairments was associated with the severity of diabetes in these patients. The issue of cognitive impairments in the co-morbidity of schizophrenia and diabetes deserves further investigation, since for schizophrenia, the prevention and treatment of diabetes might prove especially beneficial, yielding better cognitive outcomes and improved general health [17].

This study proposes to address these areas and aims to test: (1) whether cognitive impairments are different in schizophrenia with and without diabetes and different from diabetes only compared to healthy controls in the Chinese Han population; (2) whether schizophrenia with diabetes have the worst cognitive impairment among these tested groups; and (3) whether the schizophrenia with diabetes group shows the worst metabolic disorders in comparison to the other groups. We hypothesize that exacerbated cognitive deficits will be present in schizophrenia patients with diabetes relative to patients with either schizophrenia-only or diabetes-only.

## Materials and Methods

### Ethics Statement

A complete description of the study was provided to all subjects and they all provided their written informed consent to participate in the study. The protocol of the study for all subjects was approved by the Institutional Review Board, Beijing HuiLongGuan Hospital.

A psychiatrist evaluated all the participants to test whether they had the capacity to content. The research procedure was explained and it was ensured that all the research participants understood what they were required to do in the study through a detailed interview. This was aimed to maximise the understanding of the subject by using appropriate language to the subject’s level of comprehension and emotional readiness. If he/she was willing to assent to participate in the research but was unable to understand the complexity of the research processes, the research was then described to the parent or guardian with the patients at the same time. The parent and guardian would then explain the research process to the participants using methods that with gauge the subject’s interest and maximise their understanding. In these situations, a written consent was provided by the parent or guardian on behalf of the subject. If the participant did not agree to participate in the research study, they were not discriminated and were provided the same treatment as those that participated in the study.

### Subjects

A total of 182 patients with schizophrenia were recruited using a cross-sectional naturalistic design at Beijing HuiLongGuan Hospital, a Beijing City owned psychiatric hospital. The diagnoses for each patient were made by two independent and experienced psychiatrists and confirmed by the Structured Clinical Interview for DSM-IV (SCID). All patients were aged between 25 and 70 years, had schizophrenia for at least 5 years, and were on stable doses of oral antipsychotic drugs for at least 12 months prior to entry into the study. Antipsychotic treatment consisted mainly of monotherapy with clozapine (n = 82), risperidone (n = 45), and other typical antipsychotics (n = 55), including haloperidol (n = 13), chlorpromazine (n = 8), perphenazine (n = 13), sulpiride (n = 16) and others (n = 5). The mean antipsychotic dose (as chlorpromazine equivalents) was 420.7±270.2 mg/day. The average duration of the current antipsychotic treatment was 5.1±4.8 years at the time of the investigation. All patients were of the chronic type, with a mean illness course of 13.1±8.8 years. Patients were hospitalized for an average of 10.0±9.5 years. Since admission, all patients received dietetically balanced hospital meals, which were occasionally supplemented by gifts (usually fruit), and patients had the opportunity for about an hour of physical exercise every day.

One hundred and six (male/female = 65/41) outpatients with type 2 diabetes mellitus (T2DM) were recruited from the TangShan GongRen Hospital, a large general hospital located in Tangshan city, about 80 km from Beijing. They were aged 25–70 years (mean age: 54.1±8.0), with a mean duration of illness of 7.8±2.5 years (range 5–10). All the patients were receiving conventional medical treatment. The most commonly prescribed drugs were oral hypoglycemics such as Metformin and Repaglinide. No subjects used insulin. Patients with any history of diagnosed cerebral vascular disease, coronary heart disease, known neuropsychiatric or central nervous system diseases, or any other complications such as nephropathy and retinopathy of T2DM were excluded, since it is known that these cardio-cerebrovascular and central nervous system diseases are associated with cognitive impairments. Also, diabetic retinopathy and nephropathy were strongly linked to impairment in the cognitive domains [23], [24]. In addition, none of the patients displayed audiovisual or motor coordination impairment that would affect the cognitive function tests.

One hundred and ninety normal controls (male/female = 113/77) were recruited for the same period from the Beijing community. The fasting glucose levels were measured at the start of the study and again measured twice about one month later. Any normal controls with fasting glucose >6.1 mmol/l at either of the time points were excluded. Psychiatric disorders were ruled out among the controls by a psychiatric interview conducted by a psychiatrist.

Since the schizophrenia patients with diabetes were fairly old, somehow due to the diabetes focus of this study, the samples of controls and non-diabetic schizophrenia patients were specifically recruited to match the ages of the diabetes samples. First, we selected those schizophrenia patients with diabetes first, and then we selected those schizophrenia patients without diabetes matched for age, gender and education. Next, we recruited those individuals with diabetes but without schizophrenia in a general hospital based on the age, gender and education of the schizophrenia patients with diabetes. At the same time, we recruited the healthy controls without diabetes in the community that matched gender, age and education for the diabetes only group. Hence, these 4 groups were specially selected to be comparable in demographic characteristics.

We obtained a complete medical history, physical examination and laboratory tests from all subjects. All healthy controls were in good physical health. All patients were free from other physical diseases (other than T2DM), such as the central nervous system diseases of stroke, tumors, Parkinson’s disease, Huntington’s disease, seizure disorder, history of brain trauma and acute and chronic infections. Neither patients nor control subjects suffered from drug or alcohol abuse/dependence. Any subjects with a diagnosis of dementia or mild cognitive impairment (MCI) were excluded. Furthermore, all subjects were Han Chinese.

All subjects underwent fasting blood glucose testing using standard procedures. Diabetes was diagnosed as persistent fasting hyperglycemia (≥126 mg/dL) or plasma glucose levels greater than 200 mg/dL at 2 hours after a 75 g oral glucose load (2 h-PG), which is consistent with the 1999 World Health Organization diagnostic criteria for diabetes mellitus [25].

### Clinical Assessment

Two psychiatrists who had more than five years of clinical practice experience and who were blind to the clinical status and treatment conditions assessed the patient’s psychopathology using PANSS (Positive and negative syndrome scale) [26]. To ensure consistent and reliable ratings, the two psychiatrists simultaneously attended a training session for standardizing their use of the PANSS prior to the start of the study. Thereafter, they maintained an intra-class correlation coefficient of greater than 0.8 on the PANSS at repeated assessments during the course of this study.

### Cognitive Measures

The repeatable battery for the assessment of neuropsychological status (RBANS) to measure cognitive function was individually administered by trained investigators supervised by a research psychiatrist. [27] The RBANS is composed of 12 subtests that are used to calculate five age-adjusted index scores and a total score [28]. Test indices are: immediate memory (composed of list learning and story memory tasks); visuospatial/constructional (composed of figure copy and line orientation tasks); language (composed of picture naming and semantic fluency tasks); attention (composed of digit span and coding tasks); and delayed memory (composed of list recall, story recall, figure recall and list recognition tasks). Our group had previously translated RBANS into Chinese and the clinical validity and its test-retest reliability had been established among controls and schizophrenic patients [29]. To ensure consistent and reliable ratings, the two clinical psychologists simultaneously attended a training session for standardizing their use of the RBANS prior to the start of the study. Thereafter, they maintained an intra-class correlation coefficient of 0.92 on the RBANS at repeated assessments during the course of this study.

### Blood Sampling

Venous blood from a forearm vein was collected from inpatients between 7 and 9 am following an overnight fast. All fasting blood glucose, cholesterol, triglyceride, high density lipoprotein and low density lipoprotein products in the plasma were measured by a technician, who was blind to the diagnostic status of subjects. The identity of all subjects was indicated by a code number maintained by the investigator until all biochemical analyses were completed.

### Anthropometric Variables

The body weight and height were assessed in a standardized fashion to calculate Body mass index (BMI). Height was measured to the nearest millimetre, with the subjects barefooted and standing upright. Body weight was measured with an electronic scale calibrated to ±0.1 kg, and subjects were weighed in light indoor clothing. Waist circumference was measured with a measuring tape at a level midway between the lower rib margin and iliac crest with the tape in a horizontal position [30].

### Statistical Analysis

Group comparisons on the demographic and clinical variables used Chi squared or Fisher exact tests for the categorical variables and Student t-tests or analysis of variance (ANOVA) for the continuous variables. The RBANS were analysed using a 2x2 ANOVA representing the between factors of Diagnosis A (schizophrenia vs no schizophrenia) and Diagnosis B (diabetes vs no diabetes). For the RBANS comparisons, we also included age, gender and education as covariates in the multivariate analyses of covariance (MANCOVA). This was to examine significant diagnosis differences across dependent measures from the RBANS total score and its five cognitive domains, with the independent predictors being Diagnosis A, Diagnosis B and Diagnosis A multiplied by Diagnosis B interaction. Effect sizes (<0.1 =  trivial effect, 0.1–0.3 =  small effect, 0.3–0.5 =  medium effect, >0.5 =  large difference effect). were also calculated for the two-way comparisons and represented the mean difference, in standard deviation units, between the groups of interest. In the post hoc comparisons, a multiple testing correction has also been done. Multiple regression models were used to quantify the amount of variance in cognitive functioning explained by the psychopathological variables after controlling for several potential confounders, such as gender, age, education and clinical variables. SPSS version 18.0 was used to perform all statistical analysis. Data were presented as mean±SD. All *p*-values were two-tailed at the significant level of <0.05.

## Results

### Demographic and Clinical Information

Table 1 shows no significant differences among these four examined groups in age, education and gender. Schizophrenia with diabetes had the largest waist circumference. Moreover, schizophrenia with diabetes showed higher fasting glucose and triglyceride levels than schizophrenia without diabetes (both p<0.05). Compared to healthy controls, diabetes only had larger waist circumferences and fasting glucose (p<0.01). In addition, diabetes only had higher fasting glucose, triglyceride and cholesterol levels than schizophrenia with and without diabetes (p<0.001). However, there were no significant differences in height, hip, weight, BMI, high density lipoprotein or low density lipoprotein among four groups (all p>0.05).

**Table 1:** Demographic and clinical information of healthy controls, diabetes only, schizophrenia with and without diabetes.

	No schizophrenia		Schizophrenia	
	Healthy controls	Diabetes only	Schizophrenia without diabetes	Schizophrenia with diabetes
Sample size	190	106	127	55
Sex, male	59.50%	61.10%	64.60%	61.80%
Age (years)	53.1±7.4	54.1±8.0	53.3±8.3	54.4±8.1
Education (years)	9.7±3.0	9.5±3.4	9.7±2.4	9.8±2.5
Height (cm)	166.1±7.6	167.4±7.9	167.2±7.0	167.0±6.8
Waist (cm)	88.0±10.3	93.7±9.8*	92.3±14.7	104.8±10.1* +
Hip (cm)	98.0±7.2	100.9±8.5	102.8±10.0	101.4±8.5
Weight (kg)	71.3±13.5	72.4±11.2	69.5±13.8	78.5±19.8
BMI (kg/m^2^)	25.7±3.9	25.8±3.3	24.9±4.4	28.0±6.3
Fast glucose (mmol/l)	5.1±1.4	9.0±3.2*	5.2±0.5++	6.2±1.6^++#^
Cholesterol (mmol/l)	NA	6.3±2.3	4.7±0.9++	4.7±1.0++
Triglyceride (mmol/l)	NA	6.0±6.0	1.6±0.8++	2.4±1.4^++#^
High density lipoprotein (mmol/l)	NA	1.2±0.3	1.4±0.6	1.3±0.2
Low density lipoprotein (mmol/l)	NA	2.7±0.9	2.9±0.6	2.9±0.5

Mean±SD.

* indicates the comparison between healthy controls and schizophrenia with diabetes or diabetes only:

* p<0.01.

+ indicates the comparison between diabetes only and schizophrenia with or without diabetes:

+ p<0.05,

++ p<0.001.

# indicates the comparison between schizophrenia with and without diabetes:

# p<0.01. NA = not applicable.

In addition, there were no significant differences between schizophrenia with and without diabetes in any PANSS scores, duration of illness, antipsychotic types, antipsychotic dose, duration of current antipsychotic treatment and anti-Parkinsonian drug treatment (all p>0.05) (Table 2).

**Table 2:** Characteristics of schizophrenia with and without diabetes.

	Schizophrenia without diabetes	Schizophrenia with diabetes	t or x^2^	p-value
Duration of illness (years)	13.4±7.5	11.6±9.3	−0.32	0.750
Antipsychotic types (typicals/atypicals)	21/106	16/39	0.13	0.910
Antipsychotic dose (CPZ equivalents)	440.9±304.1	385.7±257.1	−1.18	0.238
Duration of current antipsychotic treatment (months)	64.2±56.3	60.4±49.3	−0.68	0.499
Anti-Parkinsonian drug (yes/no)	38/89	16/39	3.74	0.053
Score on positive symptom scale	13.1±5.8	12.3±6.0	−0.79	0.428
Score on negative symptom scale	19.9±6.6	20.8±7.6	0.83	0.408
Score general psychopathology scale	25.9±6.3	26.1±6.2	0.23	0.820
Total PANSS score	58.8±15.2	59.2±15.0	0.18	0.854

Note: Mean±SD. CPZ = chlorpromazine; PANSS = Positive and negative syndrome scale.

### Cognitive performance in schizophrenia with and without diabetes, non-schizophrenia with diabetes and healthy controls

The total and five index RBANS scores for all subjects are summarized in Table 3. After controlling for age, education and gender, the multivariate analysis of covariance (ANCOVA) revealed statistically significant differences between schizophrenia and no schizophrenia for all cognitive domains (F_1,410_ = 123.5, p<0.001). Furthermore, Diagnosis A (schizophrenia vs no schizophrenia) differences were significant for total RBANS scores and all indices (all p<0.01–p<0.001), except for the visuospatial/constructional index (p>0.05). Diagnosis B (diabetes vs no diabetes) had significant effects on total cognitive test scores and all indices (all p<0.05–p<0.001), except for the visuospatial/constructional index (p>0.05). It was also found that there were significant diagnosis A × diagnosis B effects on language (p<0.01) and delayed memory (p<0.05).

**Table 3:** The levels of cognitive function in healthy controls, diabetes only, schizophrenia with and without diabetes.

Cognitive	No schizophrenia		Schizophrenia		Diagnosis A		Diagnosis B		Diagnosis A	
index	Healthy controls (n = 190)	Diabetes only (n = 72)	Schizophrenia without diabetes (n = 127)	Schizophrenia with diabetes (n = 55)	F (*p* value)		F (*p* value)		Diagnosis B F (*p* value)	
Immediate memory	78.7±18.3	71.8±14.7	72.0±18.3	64.2±15.0	25.5	(<0.001)^a^	19.7	(<0.001)^b^	0.2	0.693
Visuospatial/construction	86.0±13.8	85.6±17.6	90.0±16.8	84.6±21.2	2.4	0.120	3.2	0.074	1.2	0.265
Language	96.3±11.0	90.1±12.9	89.6±13.2	89.4±13.9	7.4	(<0.01)^a^	5.5	(<0.05)^b^	9.9	(<0.01)^c^
Attention	89.4±18.5	87.6±17.1	85.5±15.2	80.0±15.7	16.7	(<0.001)^a^	10.2	(<0.001)^b^	0.0	0.868
Delayed memory	89.3±14.5	81.0±15.0	80.0±20.3	77.0±19.6	22.5	(<0.001)^a^	8.3	(<0.01)^b^	4.7	(<0.05)^c^
Total RBANS scores	82.9±14.1	78.1±14.0	79.2±15.8	73.6±14.1	14.9	(<0.001)^a^	15.5	(<0.001)^b^	0.7	0.388

Mean±SD. Diagnosis A (schizophrenia vs no schizophrenia).

a There were significant effects on diagnosis A. Diagnosis B (diabetes vs no diabetes).

b There were significant effects on diagnosis B.

c There were significant effects on diagnosis A × diagnosis B.

In order to decompose these two-way interactions, we compared the total and five index RBANS scores separately by healthy controls, non-schizophrenia with diabetes and schizophrenia with and without diabetes. Compared to healthy controls, non-schizophrenia with diabetes had worse cognitive performance in the immediate memory, language, attention and delayed memory (all p<0.01–p<0.001), with effect sizes ranging from 0.20–0.54. Schizophrenia with and without diabetes had worse cognitive performance in the total RBANS scores and all indices (all p<0.001), with effect sizes ranging from 0.23–0.56, except for the visuospatial/constructional index. Schizophrenia with diabetes performed worse than non-schizophrenia with diabetes in immediate memory (p<0.001) and attention (p<0.05), and they showed a trend to decreases in total RBANS scores (p = 0.069), with effect sizes ranging from 0.32–0.54. Schizophrenia with diabetes performed worse than schizophrenia without diabetes in immediate memory (p<0.01) and total RBANS scores (<0.05). They also showed a trend to decreases in attention (p = 0.052) and visuospatial/constructional functioning (p = 0.063), with effect sizes ranging from 0.37–0.45. After controlling the gender, age, education and clinical variables including duration of illness, age of onset, antipsychotic treatment (atypical vs typical, dose and duration of treatment), and hospitalization, anticholinergic drugs, as well as PANSS, both the immediate memory and overall RBANS remained significant (both p<0.05).

### Associations of cognitive impairment with clinical variables in schizophrenic patients

Since we found more significant cognitive impairment in schizophrenia compared with healthy controls, the associations of cognitive impairment with clinical psychopathological variables were examined in schizophrenic patients. Using multivariate regression analysis the following variables were independently associated with the RBANS total scores: age (beta = −0.43, t = −5.48, p<0.001); diabetes (beta = −0.18, t = −2.37, p<0.05); education (beta = 0.18, t = 2.34, p<0.05); PANSS positive symptom score (beta = 0.17, t = 2.18, p<0.05); and PANSS negative symptom score (beta = −0.17, t = −2.16, p<0.05). These factors together predicted 25.6% of the variance of the RBANS total scores.

Data for the schizophrenia with or without diabetes were analysed separately to assess the association of clinical characteristics with cognitive impairment. In schizophrenia without diabetes, multivariate regression analyses showed that the following variables were independently associated with the RBANS total score: age (beta = −0.50, t = −6.0, p<0.001); education (beta = 0.22, t = 2.75, p<0.01); PANSS positive symptom score (beta = 0.22, t = 2.0, p<0.05); and PANSS negative symptom score (beta = −0.20, t = −2.51, p<0.05). In schizophrenia with diabetes, the RBANS total score showed no multivariate associations. However, the RBANS language score was associated with: education (beta = 0.50, t = 3.70, p<0.001); PANSS general symptom score (beta = 0.33, t = 2.41, p<0.05); and cholesterol (beta = −0.30, t = −2.23, p<0.05).

## Discussion

This study examined cognitive function in healthy controls, schizophrenia with and without diabetes and diabetes only. In general, compared to healthy controls, all three patient groups displayed significant cognitive impairments. More specifically, schizophrenia with diabetes had worse cognitive functioning than schizophrenia without diabetes and diabetes only. Moreover, we found increased triglyceride levels and waist circumferences that provided significant indication of susceptibility of diabetes for schizophrenia.

In this study, we found more significant cognitive impairments in schizophrenia than in healthy controls across all cognitive domains, except visuospatial/construction. These results are consistent with our previous study [31], and are also supported by other studies [1], [4]. Studies have shown that the cognitive impairment in schizophrenia may be associated with abnormalities of brain structure, volume, neurotransmitter receptors, neuronal development and genetic mutations [32], [33], [34], [35], [36]. However, the pathophysiological mechanisms of cognitive impairments in schizophrenia are not completely understood, and deserve further investigation.

Our study indicated that cognitive impairments in diabetes only were present in immediate memory, language, attention and delayed memory. This is consistent with other studies [37], [38]. Neuropathology and functional neuroimaging studies suggest that cognitive impairments in diabetes may be caused by global and regional dysfunctions of brain energetic metabolism, especially in the hippocampal regions [39], [40], [41]. Studies have shown that chronic hyperglycemia may cause a significant loss of cortical neurons and reduce the neocortical capillary network, which is associated with cognitive deficits in diabetes [42]. Moreover, neuronal glucose malnutrition could cause excessive neuronal pruning in the brain, resulting in cognitive impairment [43]. In addition, hyperinsulinemia may inhibit synaptic activity and affect cognitive functioning [44]. Taken together, these studies suggest that diabetes may cause significant cognitive deficits.

Importantly, this study found that schizophrenia with diabetes had more serious cognitive deficits than schizophrenia without diabetes and diabetes only, mainly in immediate memory and attention. These results are consistent with a previous study which found worse cognitive performance across almost all aspects of cognition in schizophrenia with diabetes compared to schizophrenia without diabetes and diabetes only [17]. Currently, the discrepancies between Dickinson and our current study are still not clear. Some factors, such as ethnic difference, genetic background, experiment design and duration of disease may contribute to these discrepancies. Taken together, these results suggest that diabetes and schizophrenia may have additive effects on cognitive deficits, especially on immediate memory and attention.

A previous study showed that the neural underpinnings of memory, which are among the most consistent and severe neuropsychological deficits in schizophrenia, are associated with hippocampal activation [45]. Abnormalities in the hippocampal brain region may contribute to changes in learning and memory [46], [47]. Moreover, abnormalities in hippocampal structure and function, which are critically involved in cognitive impairments in learning and memory, are evident across multiple domains among schizophrenia, including brain volume [46], [48]. Furthermore, animal studies have shown that diabetes impairs hippocampus-dependent memory by reducing dendritic spine density, impairing synaptic plasticity and reducing neurogenesis in the hippocampus [40]. Human studies of type 2 diabetes mellitus also find that impaired glucose regulation contributes to hippocampal damage and to impaired memory [49]. Interestingly, these memory deficits in schizophrenia may reflect deficiencies in glucose regulation, as a few studies have shown that glucose administration improves these deficits. Other studies have reported increases in verbal declarative memory performance following glucose administration in double-blind crossover designs [50], [51]. These studies, together with our finding of impaired immediate memory in schizophrenia with diabetes, support the hypothesis that impaired glucose regulation/availability contributes to the vulnerability for memory deficits in schizophrenia, which may be associated with the extensive hippocampal abnormalities in schizophrenia.

In addition, our results showed that the interaction effects of both diagnoses on language and delayed memory were significant, suggesting that both schizophrenia and diabetes could impair the function of language and delayed memory, and produce the synergistic (multiplicative) effects on these cognitive indices.

It is worthy of mentioning that schizophrenia patients without diabetes had a higher ratio of patients who received atypical antipsychotic agents than schizophrenia patients with diabetes in our study. After controlling for the clinical variables including antipsychotic treatment (atypical vs typical, dose and duration of treatment), however, both the immediate memory and overall RBANS remained significant (both p<0.05), suggesting that the better performance in the schizophrenia without diabetes is not due to the more atypical antipsychotic drugs. However, the association between cognitive deficits and antipsychotic treatment in schizophrenia is disputable. For example, a previous study indicated that both typical and atypical antipsychotics did not have a major effect on cognitive deficits in chronic patients with schizophrenia [52]. However, other studies supported that atypical antipsychotic drugs were associated with statistically significant but small improvement in neurocognitive functioning in schizophrenia [53], [54]. Hence, this issue warrants more investigation using the longitudinal study.

Several limitations of this study should be noted. First, we did not measure the levels of glycosylated hemoglobin, which can indicate the degree of control for blood glucose in the recent two to three months [37]. Therefore, we could not confirm whether schizophrenia with diabetes had worse blood glucose control than diabetes only. Second, all patients with schizophrenia in this study were of the chronic type. We could not distinguish whether the diabetes in schizophrenia was caused by antipsychotic treatments or whether it occurred before the antipsychotic treatments. Third, we did not measure the levels of metabolite biomarkers, such as cholesterol, triglyceride, and lipoprotein in healthy controls, and thus we could not compare the healthy controls with the other groups. Fourth, we found that schizophrenia patients with diabetes had poorer cognitive function than the patients with diabetes but without schizophrenia. However, the schizophrenia patients had been hospitalized for an average of 10 years in this study. Although we did not find that there was an association between the duration of illness or hospitalization and hospitalization, it remains unclear whether long-term institutionalization may have worsened cognition function of the mentally ill patients, which warrants further investigation using a longitudinal design. Fifth, the samples were fairly old, averaging 53 or 54 years of age, with an upper age limit of 70 years. This related somehow to the diabetes focus of this study. Since the samples of controls and non-diabetic schizophrenia patients were specifically recruited to match the ages of the diabetes samples, the findings derived from such samples may not fully generalize to younger groups. Moreover, age effects, especially in the above-55 portion of each sample may confound findings somewhat. Sixth, it is noteworthy that mean age minus mean duration of illness yields an illness onset age averaging around 40 years for our schizophrenia patients. According to our previous study [55], the average onset age of the schizophrenia is around 25 years. Hence, these are not ‘typical’ schizophrenia case, probably due to the selected schizophrenia patients comorbid with diabetes, and the results will not be generalized to other schizophrenia samples. Seventh, it should be noted that our RBANS total score in normal controls in this study was nearly one standard deviation lower than the American normative data [56]. However, these results were consistent with other studies in China [29], [57], suggest that the lower RBANS total and index scores in normal population in China than in Western countries may be due to the differential education levels and cultural background. However, further research will be needed to produce normative values in Chinese population.

In summary, cognitive performance is significantly impaired in both schizophrenia and diabetes. Schizophrenia with co-morbid diabetes display increased cognitive impairment than both schizophrenia without diabetes and diabetes only, especially in immediate memory and attention, suggesting an additive effect of schizophrenia and diabetes on cognitive deficits. However, due to the nature of the cross-sectional design in our study, we cannot provide a reasonable explanation for this additive effect, although the synergism may be associated with the extensive hippocampal abnormalities caused by the abnormal glucose metabolism.
